# Individual Signatures Define Canine Skin Microbiota Composition and Variability

**DOI:** 10.3389/fvets.2017.00006

**Published:** 2017-02-06

**Authors:** Anna Cuscó, Armand Sánchez, Laura Altet, Lluís Ferrer, Olga Francino

**Affiliations:** ^1^Molecular Genetics Veterinary Service (SVGM), Veterinary School, Universitat Autònoma de Barcelona, Barcelona, Spain; ^2^Vetgenomics, Ed Eureka, Parc de Recerca UAB, Barcelona, Spain; ^3^Department of Clinical Sciences, Cummings School of Veterinary Medicine, Tufts University, North Grafton, MA, USA

**Keywords:** skin, microbiota, microbiome, dog, canine, coat, skin site, 16S

## Abstract

Dogs present almost all their skin sites covered by hair, but canine skin disorders are more common in certain skin sites and breeds. The goal of our study is to characterize the composition and variability of the skin microbiota in healthy dogs and to evaluate the effect of the breed, the skin site, and the individual. We have analyzed eight skin sites of nine healthy dogs from three different breeds by massive sequencing of 16S rRNA gene V1–V2 hypervariable regions. The main phyla inhabiting the skin microbiota in healthy dogs are Proteobacteria, Firmicutes, Fusobacteria, Actinobacteria, and Bacteroidetes. Our results suggest that skin microbiota composition pattern is individual specific, with some dogs presenting an even representation of the main phyla and other dogs with only a major phylum. The individual is the main force driving skin microbiota composition and diversity rather than the skin site or the breed. The individual is explaining 45% of the distances among samples, whereas skin site explains 19% and breed 9%. Moreover, analysis of similarities suggests a strong dissimilarity among individuals (*R* = 0.79, *P* = 0.001) that is mainly explained by low-abundant species in each dog. Skin site also plays a role: inner pinna presents the highest diversity value, whereas perianal region presents the lowest one and the most differentiated microbiota composition.

## Introduction

The skin is the living interface between an individual and the exogenous environment. It is covered with millions of microorganisms (1) interacting together with hosts’ cells and immune receptors to maintain the equilibrium (2). Bacteria are the most abundant microorganisms living on skin surface (3), and their whole population is defined as the microbiota. The high variability of the microbiota in the healthy skin has been captured during the last years using next-generation sequencing techniques [for a review, see Ref. (4)]. Marker-based approaches, mainly using 16S rRNA gene, focus on detecting who is living there—bacterial composition and diversity.

Main phyla inhabiting human skin are Actinobacteria, Firmicutes, Bacteroidetes, and Proteobacteria. A feature of human cutaneous microbiota is the existence of different microhabitats, which are characterized by the predominance of a specific taxa: sebaceous sites (occiput, glabella, alar crease, and manubrium) with *Propionibacterium* spp; moist sites (nare, axilla, and inguinal crease) with *Staphylococcus* and *Corynebacterium* spp; and dry sites (palms and butlock) with gram-negative microorganisms (5). According to the first extensive study reported, dogs harbor mainly the same phyla as human skin (6). Fusobacteria was also detected as a main phylum, when only considering the paws and the forehead (7) and also in a recent study considering the groins (8). In humans, the variation is higher among different microhabitat skin sites of the same individual than among skin sites from the same microhabitat in different individuals (5, 9). Several differences among skin sites have been described in dogs (6), but to our knowledge, no microhabitats have been defined.

Different factors such as the environment, host genetic variation, lifestyle, or hygiene cause shifts on the microbial communities of the skin (10). These shifts on the microbiota structure and composition could establish a dysbiotic state, which if not recovered could result on a dermatologic affliction. Dysbiosis of the skin microbiota has been associated with several skin afflictions in humans, such as atopic dermatitis (11, 12), psoriasis (13, 14), and acne vulgaris (15). In canine microbiota studies, association between atopic dermatitis and microbiota has been assessed showing less richness on affected animals, either when considering bacteria (6, 16) or fungal communities (17). However, in allergen-induced canine atopic dermatitis, no significant differences on diversity were reported (8). Moreover, recent studies have reported significant increases of *Staphylococcus* and *Corynebacterium* in dogs with this disease (8, 16). Nevertheless, a better characterization of the cutaneous microbiota of healthy dogs seems to be necessary before understanding its role in disease conditions.

There is much less knowledge about the potential functions of the mammals’ microbiota. The potential function of a bacterial community can be assessed either directly, using shotgun metagenomics, or indirectly, using 16S data and a predictive software such as Phylogenetic Investigation of Communities by Reconstruction of Unobserved States (PICRUSt) (18). Langille et al. used this tool with the Human Microbiome Project dataset (19) obtaining sufficiently accurate results, even for skin samples (18). In canine intestinal microbiota studies, shotgun metagenomics has been used to study microbiota variability when feeding animals with two different diets (20), and PICRUSt was used in dogs suffering idiopathic inflammatory bowel disease (21). To our knowledge, no studies have assessed potential functions of the microbiota at the skin level.

Our aim was to characterize the composition and variability of the skin microbiota on healthy dogs, considering the breed—specially the hair coat—the skin site, and the individual. We sampled nine healthy dogs from three breeds representing the diversity of canine hair coats: French Bulldog (FB; short hair), German Shepherd (GS; long hair with undercoat), and West Highland White Terriers (WHs; wired hair) (22). These three breeds were also selected because they are among the most predisposed to suffer from atopic dermatitis (23). We also aimed to predict the functional profile of the microbiota of different skin sites using PICRUSt.

## Materials and Methods

### Ethics Statement

The dogs in the study were examined during routine veterinary procedures by the veterinary clinics participating in the study. All samples were collected and used in the study with verbal owner consent. As the data are from client-owned dogs that underwent normal preventative veterinary examinations, there was no “animal experiment” according to the legal definitions in Spain, and approval by an ethical committee was not necessary.

### Individuals Included and Sample Collection

**Figure 1:**
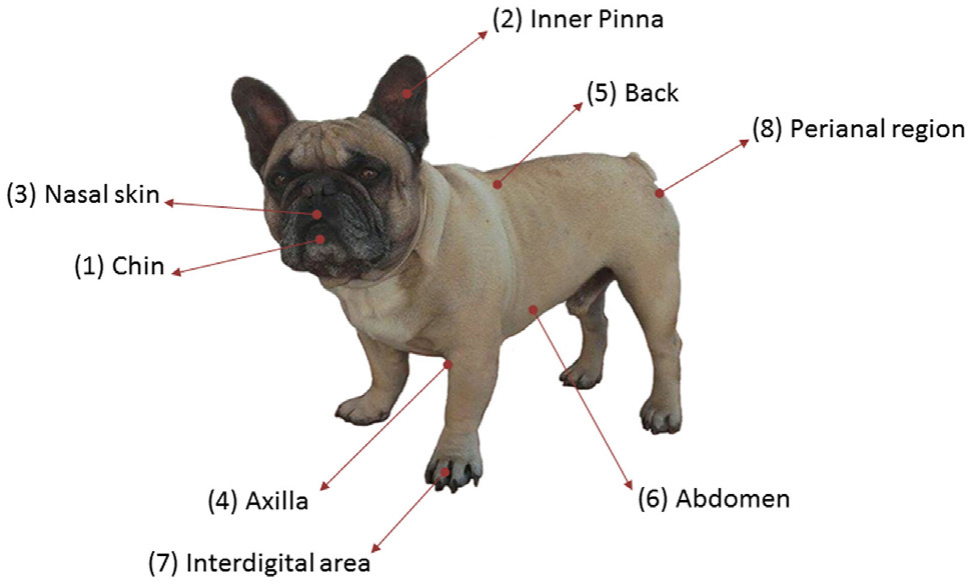
**Skin sites sampled per dog**.

A cross-sectional study was performed in nine healthy dogs to analyze skin microbiota variability in several skin sites, considering the breed, the hair coat, and the individual. They were all pure-breed dogs ranging from 3 months to 12 years of age and from different households visiting the veterinary clinic for routine procedures (Table S1 in Supplementary Material). All of them lived in urban or periurban environment. Samples from three FBs (FB1, FB2, and FB3), three GSs (GS1, GS2, and GS3), and three West Highland WHs (WH1, WH2, and WH3) were included. Skin samples were collected from eight skin regions: chin, inner pinna, nasal skin, axilla, back, abdomen, interdigital area, and perianal region. These regions are named as 1, 2, 3, 4, 5, 6, 7, and 8, respectively (Figure 1). Samples were obtained by firmly rubbing each area using Sterile Catch-All™ Sample Collection Swabs (Epicentre Biotechnologies) soaked in sterile SCF-1 solution (50 mM Tris buffer (pH = 8), 1 mM EDTA, and 0.5% Tween-20). To minimize sample cross-contamination, the person sampling wore a fresh pair of sterile gloves for each individual. Swabs were stored at 4°C until DNA extraction, within the following 24 h.

### DNA Extraction

Bacterial DNA was extracted from the swabs using the PowerSoil™ DNA isolation kit (MO BIO) under manufacturer’s conditions, with one modification. At the first lysis step, the swab tip with the sponge was cut and introduced in the beads’ tube, until the first transference of the supernatant to a new tube. The remaining steps were performed as described by the manufacturer. DNA samples (100 µl) were stored at −20°C until further processing.

To assess for contaminations from the laboratory or reagents, a sterile swab tip was processed in the same conditions as the skin microbiota samples, giving negative results.

### PCR Amplification and Massive Sequencing

V1–V2 regions of 16S rRNA gene were amplified using the widely used primer pair F27 (5′-AGAGTTTGATCCTGGCTCAG-3′) and R338 (5′-TGCTGCCTCCCGTAGGAGT-3′). PCR mixture (50 uL) contained 5 µl of DNA template (~5 ng), 5 µl of 10× AccuPrime™ PCR Buffer II, 0.2 µM of each primer, and 1 U of AccuPrime™ Taq DNA Polymerase High Fidelity (Life Technologies). The PCR thermal profile consisted of an initial denaturation for 2 min at 94°C, followed by 30 cycles for 1 min at 94°C, 1 min at 55°C, 1 min at 72°C, and a final step for 7 min at 72°C. To assess possible reagent contamination, each PCR reaction included a no template control sample, which did not amplify. For each amplicon, quality and quantity were assessed using Agilent Bioanalyzer 2100 and Qubit™ fluorometer. Both primers included sequencing adaptors at the 5′ end, and forward primers were tagged with different barcodes to pool samples in the same sequencing reaction. Each pool contained 8 barcoded samples. A total of 9 pools were sequenced on an Ion Torrent Personal Genome Machine (PGM) with the Ion 318 Chip Kit v2 and the Ion PGM™ Sequencing 400 Kit (Life Technologies) under manufacturer’s conditions. The raw sequences have been deposited in NCBI under the Bioproject accession number PRJNA357691.

### Quality Control of the Sequences and Operational Taxonomic Unit (OTU) Picking

Raw sequencing reads were demultiplexed, quality filtered, and analyzed using QIIME 1.9.1 (24). Reads included had a length greater than 300 bp; a mean quality score above 25 in sliding window of 50 nucleotides; no mismatches on the primer; and default values for other quality parameters. Quality-filtered reads were clustered into OTUs at 97% similarity, using UCLUST (25) in an open reference approach for taxonomy analyses and a closed reference approach for functional profiling. Taxonomic assignment of representative OTUs was performed using the RDP Classifier (26) against Greengenes v13.8 database (27). Alignment of sequences was performed using PyNast (28) as default in QIIME pipeline. Chimera checking was performed using Chimera Slayer (29).

We applied two extra filtering steps in aligned and taxonomy-assigned OTU table. First, sequences that belonged to chloroplasts class were filtered out. After that, sequences representing less than 0.005% of total OTUs were also filtered out [as previously done in Ref. (30)] from the chloroplast filtered OTU table. After these two extra filtering steps, we lost a mean of 27% of sequences (median of 25%, ranging from 2 to 77%) and a mean of 21% of sequences (median of 15%, ranging from 1 to 77%) in open and closed reference approaches, respectively (Data Sheet S1 in Supplementary Material).

### Downstream Bioinformatics Analyses: Diversity, Composition, Potential Functions, and Statistical Tests

Downstream analyses were performed using QIIME 1.9.1 (24) with the filtered OTU table. Reads are clustered against a reference sequence collection, and all of the reads that do not hit a sequence in the reference sequence collection are excluded from downstream analyses in a closed reference approach or are subsequently clustered *de novo* in an open reference approach. To standardize samples with unequal sequencing depths, analyses were performed using random subsets of 25,000 sequences per sample in the open reference approach and random subsets of 10,000 sequences per sample in the closed reference approach. The perianal sample of one FB (FB1.8) failed this parameter and was discarded for posterior analyses.

Alpha diversity analysis assesses the diversity within a sample. In alpha diversity, we used two different metrics: observed species to assess richness and Shannon index to assess evenness. We assessed statistical significance with 999 permutations using the non-parametric Monte Carlo permutation test and corrected the *P* value through false discovery rate.

To assess the differences in the alpha diversity and composition at the individual level, we collapsed the eight skin samples from a dog using QIIME v1.9.1 to form a unique sample representing the individual. Therefore, the sample size for analyzing the individual effect is nine.

To assess the differences in the alpha diversity and composition when considering the breed, we used two approaches: (A) analyzing each skin site independently and (B) using the QIIME collapsed values from the eight skin site samples for each dog (*n* = 9). In the first approach, we group the three samples corresponding to a skin site from a breed and assessed differences in breeds per skin site; e.g., GS1.1, GS2.1, and GS3.1 as GS_chin and we compared them to FB_chin and WH_chin. In the second approach, we group the three collapsed individual samples from each breed; e.g., GS1, GS2, and GS3 as GS and we compared them to FB and WH.

Beta diversity analysis assesses the similarities among samples of the same community. Beta diversity was performed using both weighted and unweighted UniFrac distance metrics (31). Weighted UniFrac considers phylogeny, taxa, and relative abundances, whereas unweighted UniFrac only considers phylogeny and taxa. Those distance matrices were used to create PCoA plots and unweighted pair group method with arithmetic mean (UPGMA) trees. Trees were plotted using FigTree (REF). ANOSIM and adonis statistical methods were applied to evaluate if some variables were determining grouping and to which extent.

PICRUSt (18) was used to predict the functional profile of skin bacterial communities using 16S rRNA gene data obtained using a closed reference approach in QIIME v1.9.1. Kyoto Encyclopedia of Genes and Genomes (KEGG) (32) Ortholog (KO) hierarchy was used to make inferences of the functional gene content.

Linear discriminant analysis (LDA) effect size (LEfSe) (33) was used to compare groups and to identify differentially abundance distribution in both taxa and predicted functions (α = 0.05 and with an LDA score >3.0).

## Results

To assess variability and composition of dog skin microbiota, we performed a cross-sectional study with healthy dogs from three breeds. We have analyzed 72 samples from 9 dogs: 3 FBs, 3 GSs, and 3 West Highland WH. We sampled eight skin sites: chin, inner pinna, nasal skin, axilla, back, abdomen, interdigital region, and perianal area, which are named as 1, 2, 3, 4, 5, 6, 7, and 8, respectively (Figure 1). These anatomic sites were selected to represent the regional diversity of the canine skin (34).

We found a total of 2,092 bacterial OTUs living on dog skin, which were taxonomically classified into 20 phyla, 51 classes, 69 orders, 132 families, and 245 genera. Data Sheet S2 in Supplementary Material contains several OTU tables: the complete OTU table for the 72 samples, the OTU table at family level obtained for all the samples, the OTU table collapsed by site, and the OTU table collapsed by individual.

**Figure 2:**
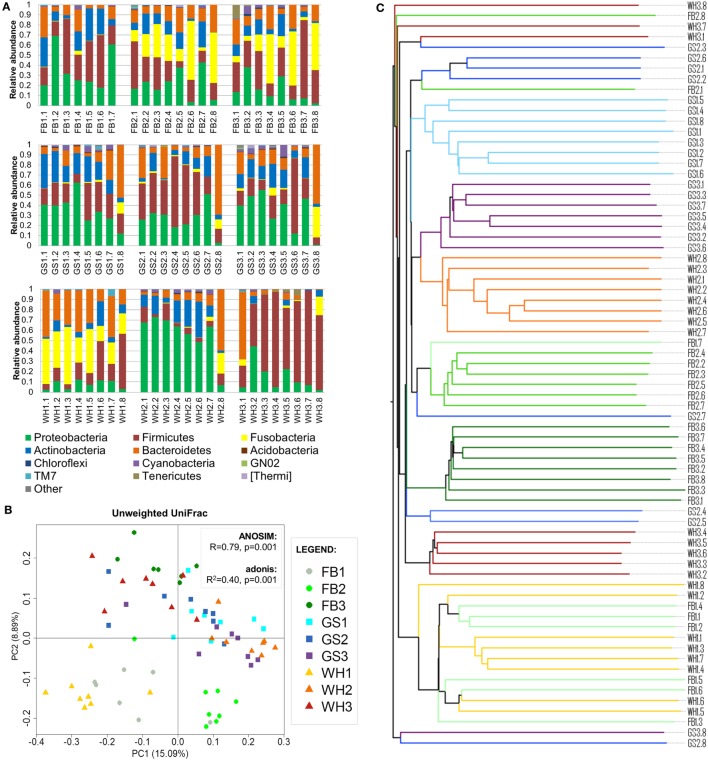
**Individual signatures on dog skin**. **(A)** Taxonomy bar plot of each sample at phylum level. The first two letters with the number represent the breed and individual; and the numbers represent each skin site. **(B)** PCoA plot using unweighted UniFrac metrics colored by individual with values of ANOSIM and adonis statistical tests. **(C)** Unweighted pair group method with arithmetic mean tree associated with **(B)**; branches are colored by individual.

The abundances of the main phyla differed on each sample (Figure 2A). The main phyla on skin samples were Proteobacteria (1–73%), Firmicutes (3–93%), Fusobacteria (0–58%), Bacteroidetes (0–69%), and Actinobacteria (0–35%), followed by Cyanobacteria, Tenericutes, TM7, and others with lower abundances.

Alpha diversity values were also very variable among samples (Data Sheet S3 and Figure S1 in Supplementary Material). The richness (observed species) ranged from 145.6 in the chin of WH1 to 928.8 in the inner pinna of GS1 (average of 488.42). The evenness (Shannon Index) ranged from 0.959 in the axilla of WH3 to 8.559 in the abdomen of WH2 (average of 5.8).

To assess if the variability of the dog skin microbiota depended on individual, breed, and/or skin site and to which extent, we clustered the samples using UPGMA trees and assessed statistical significance using adonis and ANOSIM tests (Figures 2B,C). We found that the main force driving the variability in dog skin microbiota composition is the individual, followed by the skin site and the breed.

Despite the high variability detected among samples, all of them were skin microbiota of healthy dogs and in fact shared some of their taxonomy. Thus, to assess the homogeneity of the samples, we analyzed the core microbiota. To complete the analysis, we assessed the potential functions of the bacterial community using PICRUSt.

### Individual

Samples from the same individual tended to cluster together (Figures 2B,C). Statistical analysis using adonis test confirmed this result: the clustering of samples per individual significantly explained 40% (unweighted UniFrac) and 45% (weighted UniFrac; Figure S2A in Supplementary Material) of the distances among samples. Moreover, ANOSIM *R* value was close to +1 (*R* = 0.79, *P* = 0.001) in unweighted UniFrac, suggesting a strong dissimilarity among groups that was mainly explained by low-abundant species in each dog. Therefore, the individual was the variable that explained most differences among samples.

Seven of nine dogs had a taxonomic profile with the main bacterial phyla: Firmicutes and Proteobacteria with higher abundances than Actinobacteria or Bacteroidetes (Figure 2A). From these dogs, FB2, FB3, and WH1 presented also Fusobacteria as one of the main phyla if not the greatest one, whereas in GS1, GS2, GS3, and FB1, this phylum was almost absent. Two of nine dogs presented a predominant phylum (>50% of the total abundance) over the others, WH2 with Proteobacteria and WH3 with Firmicutes. The abundances of these two phyla and others were differentially distributed (Figure S2B in Supplementary Material) (α = 0.05, LDA score >3).

The abundances differed in each individual, not only at the phylum level but also at the deeper taxonomic levels, such as family level (Data Sheet S2 in Supplementary Material). We can detect some individual-specific families, when looking at the most abundant families (Table 1): *Listeriaceae* representing a 22.5% of total microbiota composition for GS2; *Porphyromonadaceae* with a 26.1% for WH1; and or *Enterobacteriaceae* with a 12% for FB1. On the other hand, *Streptococcaceae* was present in all the individuals with low percentages, in exception of WH3 with 59% of the total composition that making it the individual with the lowest evenness value (3.71 of Shannon Index, Data Sheet S3 in Supplementary Material). Depending on the individual, families representing more than 5% (Table 1) were describing from 36.3 to 78.6% of total microbiota composition.

**Table 1:** **Skin microbiota composition at family level for each individual**.

Phylum	Family	FB1	FB2	FB3	GS1	GS2	GS3	WH1	WH2	WH3
Proteobacteria	*Rhodospirillaceae*	0.4%	1.5%	1.7%	2.4%	1.2%	5.5%	0.2%	10.9%	0.5%
Proteobacteria	*Sphingomonadaceae*	2.5%	3.3%	1.5%	3.0%	1.2%	1.9%	0.7%	9.0%	0.7%
Proteobacteria	*Enterobacteriaceae*	12.0%	0.2%	0.1%	0.2%	0.3%	0.1%	0.1%	0.0%	0.2%
Proteobacteria	*Pasteurellaceae*	6.4%	1.2%	0.1%	0.2%	0.9%	0.4%	0.4%	0.1%	0.2%
Proteobacteria	*Moraxellaceae*	0.8%	0.2%	0.3%	0.3%	2.0%	0.4%	0.5%	6.6%	0.4%
Firmicutes	*Listeriaceae*	0.1%	0.0%	0.0%	0.1%	22.5%	0.5%	0.2%	0.1%	0.0%
Firmicutes	*Staphylococcaceae*	10.2%	4.6%	7.0%	5.6%	1.2%	10.6%	0.8%	3.9%	0.3%
Firmicutes	*Streptococcaceae*	2.2%	2.6%	1.1%	1.0%	3.3%	1.6%	0.2%	0.1%	59.1%
Firmicutes	*Clostridiaceae*	0.5%	0.5%	8.2%	4.9%	1.4%	1.6%	0.9%	0.4%	2.6%
Firmicutes	*Lachnospiraceae*	0.4%	0.3%	6.9%	1.8%	0.6%	1.0%	2.9%	0.9%	0.7%
Fusobacteria	*Fusobacteriaceae*	0.6%	21.2%	23.9%	1.9%	2.3%	5.4%	32.2%	3.4%	3.6%
Fusobacteria	*Leptotrichiaceae*	0.3%	5.9%	0.0%	0.1%	0.1%	0.1%	0.0%	0.0%	0.0%
Bacteroidetes	*Bacteroidaceae*	0.4%	3.1%	6.6%	6.2%	9.0%	8.6%	1.4%	5.7%	0.3%
Bacteroidetes	*Porphyromonadaceae*	1.8%	1.6%	3.3%	0.2%	4.7%	1.0%	26.1%	1.4%	8.5%
Bacteroidetes	*Weeksellaceae*	5.4%	0.8%	0.3%	0.8%	3.5%	1.8%	1.7%	1.0%	0.2%
Actinobacteria	*Corynebacteriaceae*	4.8%	0.5%	1.4%	6.7%	1.0%	2.6%	2.1%	1.4%	1.3%
Actinobacteria	*Intrasporangiaceae*	5.2%	1.4%	0.4%	0.9%	0.3%	2.6%	2.5%	0.3%	0.0%
**% of microbiota explained by taxa >5%**		**53.9%**	**48.9%**	**62.8%**	**36.3%**	**55.6%**	**45.6%**	**72.6%**	**45.0%**	**78.6%**

*List of the taxa that represent >5% of the total microbiota composition and their abundances considering the individual*.

### Skin Site

**Figure 3:**
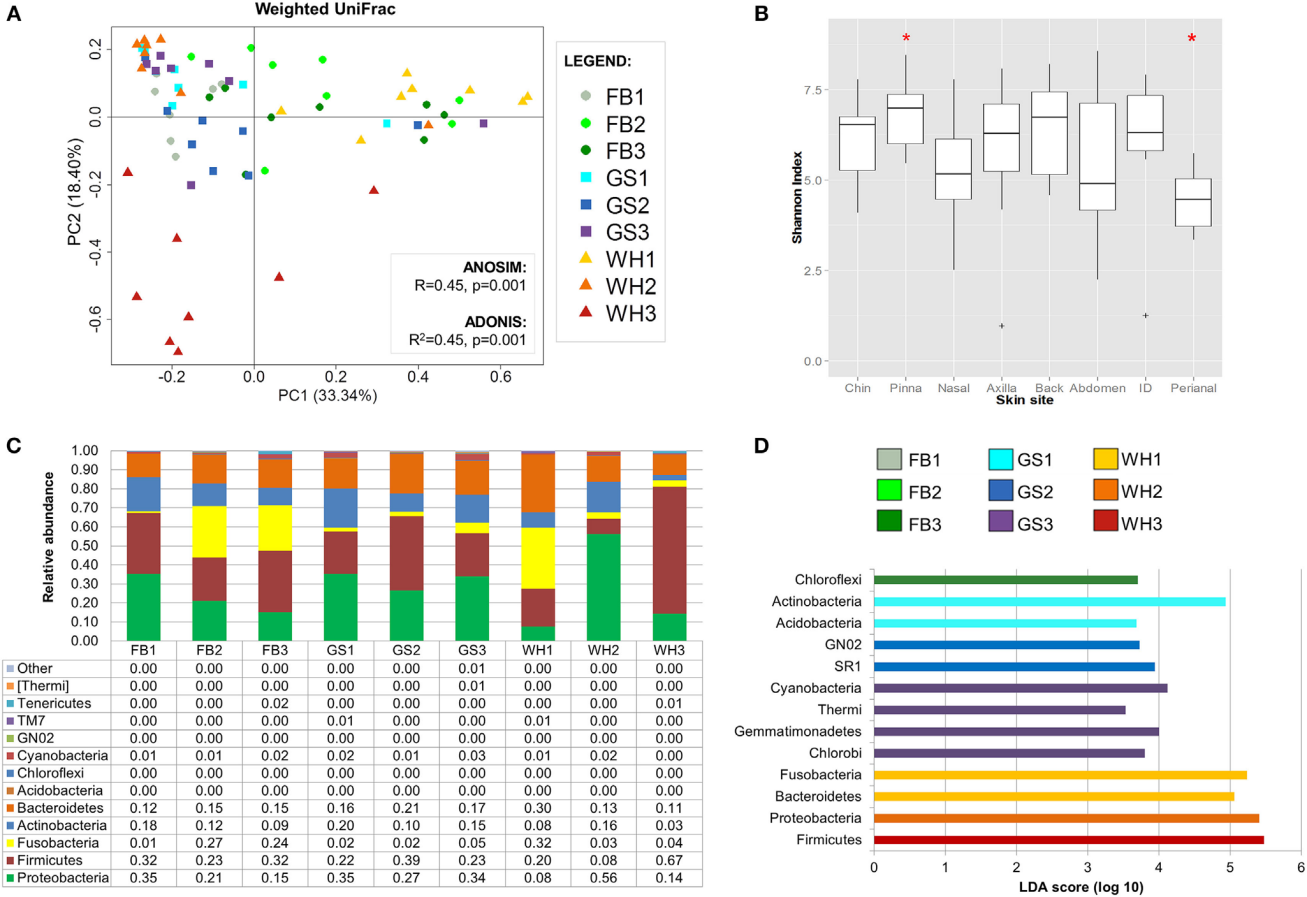
**Dog skin microbiota analysis considering site**. **(A)** PCoA plot using weighted UniFrac metrics colored by skin site with values of ANOSIM and adonis statistical tests. Perianal region is circled in brown. **(B)** Boxplots of alpha diversity values. Marked with a red asterisk the two comparisons that were statistically different when using Monte Carlo permutation test (*P* < 0.05). **(C)** Bar plot representing skin microbiome composition at phylum level per skin site; each bar represents the mean values of the nine dogs per each skin site. **(D)** Histogram of linear discriminant analysis (LDA) effect size scores for differentially abundance distribution (α = 0.05, LDA score >3) of bacterial phyla and classes among individuals.

Clustering samples per skin site significantly explained 19% of the distances, when considering composition, phylogeny, and relative abundances (weighted UniFrac; Figure 3A). Visually inspecting the beta diversity plot, we found that perianal samples cluster together.

Inner pinna presented the highest diversity value with an average richness of 610.82 observed species and an average evenness of 6.85 of Shannon index, whereas the perianal region presented the lowest diversity, with only 323.1 observed species and a Shannon index of 4.41 (Data Sheet S3 in Supplementary Material). These two skin regions were significantly different between each other when considering evenness (Figure 3B; *P* = 0.028).

Most of the skin sites had Proteobacteria and Firmicutes as the most abundant phyla, adding up to more than 55% of the total microbiota composition (Figure 3C). Chin had also Bacteroidetes as an abundant phylum with a 14.6% of *Porphyromonadaceae*, becoming the main family of this skin site. The perianal region was the exception and presented the most different composition profile (Figure 3 and Table 2; Data Sheet S2 in Supplementary Material). In perianal region, Bacteroidetes was the main phylum, followed by Firmicutes and Fusobacteria. Moreover, Proteobacteria, which is one of the main phyla inhabiting dog skin, was almost absent. The three main families inhabiting perianal region were *Bacteroidaceae* with 32.5% (Bacteroidetes), *Fusobacteriaceae* with 25.6% (Fusobacteria), and *Lachnospiraceae* with 6.4% (Firmicutes).

**Table 2:** **Skin microbiota composition at family level for each skin site**.

Phylum	Family	Chin	Inner pinna	Nasal skin	Axilla	Back	Abdomen	Interdigital	Perianal
Proteobacteria	*Rhodospirillaceae*	2.3%	6.6%	2.9%	3.1%	3.0%	0.8%	2.9%	0.1%
Proteobacteria	*Neisseriaceae*	4.9%	1.3%	3.3%	1.5%	0.6%	1.1%	5.1%	0.0%
Proteobacteria	*Enterobacteriaceae*	0.3%	6.7%	2.1%	0.6%	0.2%	0.1%	0.1%	0.5%
Proteobacteria	*Moraxellaceae*	1.0%	0.2%	6.7%	0.6%	0.5%	0.6%	0.3%	0.1%
Firmicutes	*Listeriaceae*	1.7%	2.9%	0.2%	6.9%	5.7%	2.7%	0.8%	0.1%
Firmicutes	*Staphylococcaceae*	4.5%	2.2%	12.9%	1.7%	5.1%	9.6%	1.6%	0.5%
Firmicutes	*Streptococcaceae*	5.2%	4.3%	10.6%	11.1%	6.2%	9.5%	11.0%	5.8%
Firmicutes	*Clostridiaceae*	1.6%	2.6%	1.1%	0.8%	1.5%	1.4%	5.4%	4.6%
Firmicutes	*Lachnospiraceae*	1.1%	0.7%	0.6%	0.8%	1.0%	0.7%	3.0%	6.4%
Fusobacteria	*Fusobacteriaceae*	7.8%	6.7%	11.6%	10.6%	7.4%	13.5%	3.5%	25.6%
Bacteroidetes	*Bacteroidaceae*	0.4%	0.2%	0.4%	3.1%	0.6%	2.5%	0.5%	32.5%
Bacteroidetes	*Porphyromonadaceae*	14.6%	3.3%	7.3%	5.2%	4.4%	2.8%	4.3%	1.3%
Bacteroidetes	*Weeksellaceae*	6.0%	0.8%	2.4%	0.8%	0.6%	1.1%	1.5%	0.0%
Actinobacteria	*Corynebacteriaceae*	6.2%	2.6%	1.2%	2.0%	1.8%	1.6%	0.9%	3.1%
**% of microbiota explained by taxa >5%**		**57.5%**	**41.2%**	**63.3%**	**48.8%**	**38.3%**	**48.1%**	**40.8%**	**80.6%**

*List of the taxa that represent >5% of the total microbiota composition and their abundances considering the skin site*.

We detected differentially distributed abundances on skin sites with LEfSe analyses (α = 0.05, LDA score >3) at the phylum and class level (Figure 3D) and up to the family level (Figure S3 in Supplementary Material). At the phylum and class level, Proteobacteria was significantly overrepresented at inner pinna, mainly due to the members of Alphaproteobacteria class; and Actinobacteria were overrepresented at the back. Some of the lowest abundant phyla were significantly more represented in a specific skin site: GN02 and TM7 at chin and [Thermi] (mainly from the *Deinococci* class) at inner pinna.

### Breed

The breed explained fewer differences among the samples, but it did explain some differences. Clustering samples per breed significantly explained 10% (unweighted UniFrac) and 9% (weighted UniFrac) of the distances among samples (Figures S4A,B in Supplementary Material).

To assess the effect of the breed in diversity and composition, we used two approaches: (A) analyzing each skin site separately considering the breed and (B) analyzing each collapsed dog sample per breed, adding up together all the values of the eight skin sites to form an individual dog value and grouping the three dogs from the same breed.

At taxonomic composition level, when analyzing each skin site per breed, we saw some differences (α = 0.05, LDA score >3) (Figure S4C in Supplementary Material). At phylum level, Tenericutes were overrepresented at nasal skin of FB. At family level, GS had an overrepresentation of *Dermabacteraceae* at axilla and *Corynebacteriaceae* and *Williamsiaceae* at the interdigital region, whereas FB had *Burkholderiaceae* and *Bacillaceae* at axilla, *Gemellaceae* at the interdigital region, and *Gordoniaceae* at back and chin. When collapsing all the eight skin sites to obtain an individual sample, we only detected three families with differentially distributed abundances: *Sphingobacteriaceae* and *Dermabacteraceae* in GS and *Enterococcaceae* in FB (Figure S4D in Supplementary Material). All of these taxa had low relative abundances (Data Sheet S2 in Supplementary Material).

In alpha diversity analysis, we detected no statistical differences, both when analyzing each skin site separately (Figure S5A in Supplementary Material) and when analyzing the collapsed dog samples (Figure S5B in Supplementary Material).

### Core Skin Microbiota

Each dog had its own microbiota profile, but there were also taxa shared among all samples even at low-abundant level, which we can define as the skin *core* microbiota of our cohort of individuals.

Families found in all the skin samples analyzed in this study were *Corynebacteriaceae* (Actinobacteria); *Streptococcaceae* and *Lachnospiraceae* (Firmicutes); *Fusobacteriaceae* (Fusobacteria); and *Comamonadaceae, Oxalobacteraceae*, and *Neisseriaceae* (Proteobacteria) (Table 3).

**Table 3:** **Skin core microbiota at family level for each individual and skin site**.

Phylum	Family	GS1	GS2	GS3	FB1	FB2	FB3	WH1	WH2	WH3
Actinobacteria	*Corynebacteriaceae*	6.7%	1.0%	2.6%	4.8%	0.5%	1.4%	2.1%	1.4%	1.3%
Firmicutes	*Streptococcaceae*	1.0%	3.3%	1.6%	2.2%	2.6%	1.1%	0.2%	0.1%	59.1%
	*Lachnospiraceae*	1.8%	0.6%	1.0%	0.4%	0.3%	6.9%	2.9%	0.9%	0.7%
Fusobacteria	*Fusobacteriaceae*	1.9%	2.3%	5.4%	0.6%	21.2%	23.9%	32.2%	3.4%	3.6%
Proteobacteria	*Comamonadaceae*	3.3%	1.5%	1.8%	1.6%	1.1%	0.6%	0.9%	1.9%	0.8%
	*Oxalobacteraceae*	2.8%	3.4%	2.2%	1.1%	0.9%	0.5%	0.6%	3.5%	3.9%
	*Neisseriaceae*	1.6%	3.9%	1.5%	4.6%	0.8%	1.0%	1.9%	4.9%	0.4%
**% of microbiota explained by core taxa**		**19.1%**	**16.1%**	**16.2%**	**15.3%**	**27.5%**	**35.5%**	**40.7%**	**16.0%**	**69.9%**

**Phylum**	**Family**	**Chin**	**Inner pinna**	**Nasal skin**	**Axilla**	**Back**	**Abdomen**	**Interdigital**	**Perianal**	

Actinobacteria	*Corynebacteriaceae*	6.2%	2.6%	1.2%	2.0%	1.8%	1.6%	0.9%	3.1%	
Firmicutes	*Streptococcaceae*	5.2%	4.3%	10.6%	11.1%	6.2%	9.5%	11.0%	5.8%	
	*Lachnospiraceae*	1.1%	0.7%	0.6%	0.8%	1.0%	0.7%	3.0%	6.4%	
Fusobacteria	*Fusobacteriaceae*	7.8%	6.7%	11.6%	10.6%	7.4%	13.5%	3.5%	25.6%	
Proteobacteria	*Comamonadaceae*	1.7%	1.4%	1.9%	2.0%	1.9%	1.8%	1.2%	0.1%	
	*Oxalobacteraceae*	1.5%	3.2%	1.6%	2.0%	2.6%	2.1%	3.6%	0.2%	
	*Neisseriaceae*	4.9%	1.3%	3.3%	1.5%	0.6%	1.1%	5.1%	0.0%	
**% of microbiota explained by core taxa**		**28.4%**	**20.2%**	**30.8%**	**30.0%**	**21.4%**	**30.4%**	**28.2%**	**41.2%**	

*List of the taxa shared in all samples included in the study, their abundances, and distributions by individual and skin site*.

The skin core microbiota at family level explained from 15.3 to 40.7% of the individual composition and from 20.2 to 41.2% of the skin site composition. It reached 69.9% for WH3 (with 59.1% of *Streptococcaceae*). Although a group of families constituted the core microbiota, their abundances were specific for each individual and site.

When we consider that the skin core microbiota is defined by taxa present in 85% of the samples (61 of 71 samples; to exclude some specific site or specific individual), the skin core microbiota explained a mean of 78% of the skin composition at both the individual and the skin site level, and we found 39 different families (Data Sheet S4 in Supplementary Material).

### Predicted Functions

We used 16S rRNA gene sequencing data to predict the functional profile of dog skin microbiota samples, applying PICRUSt. PICRUSt developers (18) and more recently Meisel et al. (35) reported strong correlations between human metagenomic data sets and 16S-based functional prediction in skin microbiota.

We found up to 41 predicted functions for the dog skin microbiota, when considering the second level of KO hierarchy. Membrane transport (environmental information processing); replication and repair (genetic information processing); and amino acid, carbohydrate, and energy metabolism (metabolism) are the functions more spread and represented, with a mean relative abundance of 12, 7.9, 10.3, 10.4, and 5.6%, respectively (Data Sheet S6 in Supplementary Material).

**Figure 4:**
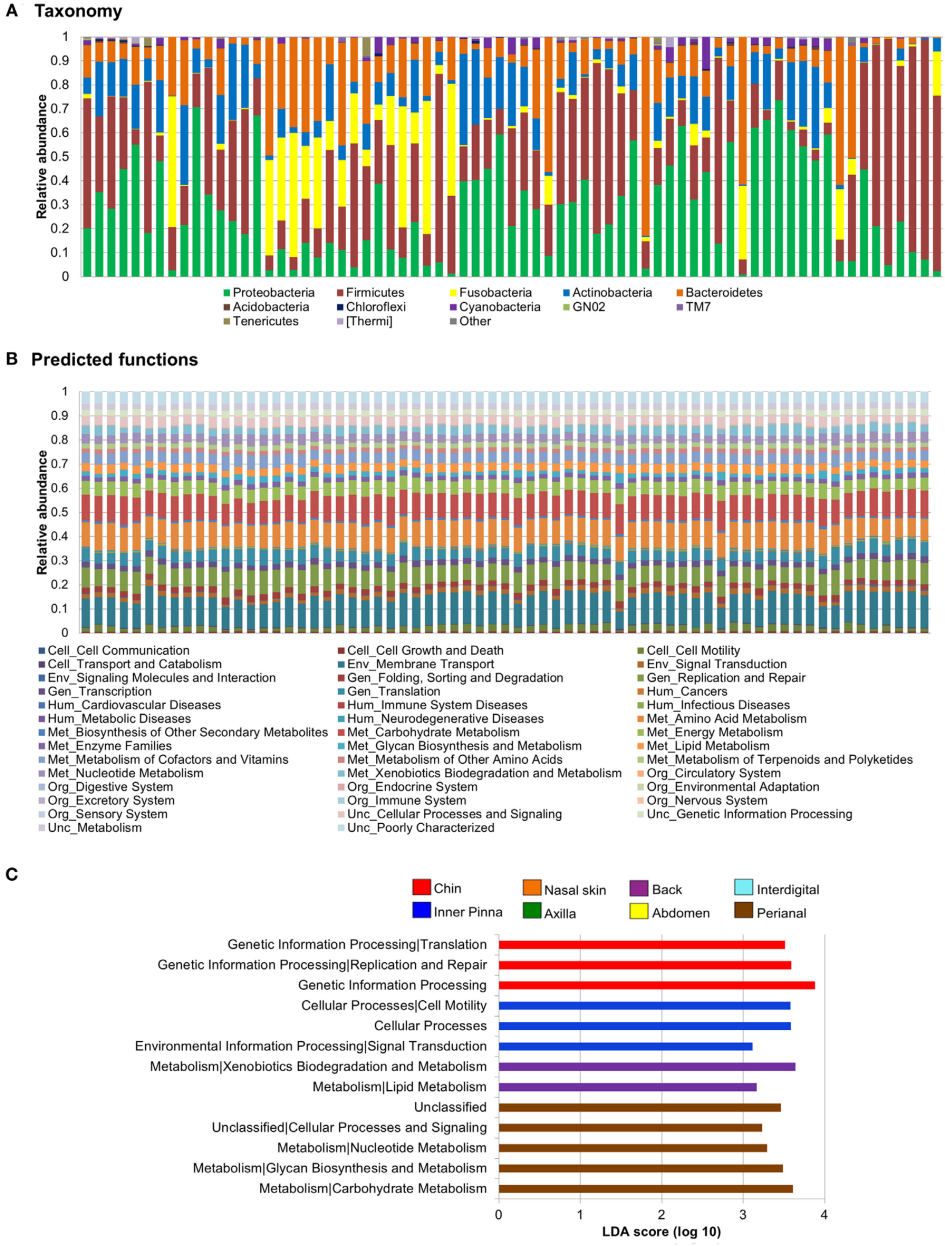
**Skin microbiota relative abundances: taxa vs predicted functions**. Bar plots obtained through a closed reference approach to be comparable between them (see Section “Materials and Methods”). Each stacked bar represents relative abundances of each sample included in the study. Relative abundances of **(A)** bacteria at phylum level and **(B)** predicted functions (second level of the Kyoto Encyclopedia of Genes and Genomes Ortholog hierarchy) based on Phylogenetic Investigation of Communities by Reconstruction of Unobserved States data set. **(C)** Histogram of linear discriminant analysis (LDA) effect size scores for differentially abundance distribution (α = 0.05, LDA score >3) of predicted functions. Complete list of predicted functions on dog skin and their relative abundances is available in Data Sheet S6 in Supplementary Material.

Taxa composition profiles became more uniform when converting them to predicted functions (Figures 4A,B). However, we found some differentially distributed abundances in predicted functions at breed, individual, and skin site level (α = 0.05, LDA score >3). We focused on assessing differences on the functional prediction among skin sites (Figure 4C).

Some predicted functions were overrepresented in back, chin, perianal region, and inner pinna and differentially distributed from all other sites. In pinna, we found overrepresentation of cellular processes and cell motility (cellular processes) and also signal transduction (environmental information processing). In perianal region, three metabolism pathways were increased: carbohydrate metabolism, glycan biosynthesis and metabolism, and nucleotide metabolism. In chin, we found overrepresentation of genetic information processing and its sublevel pathways—replication and repair and translation. In the back, three metabolism pathways were increased: xenobiotics biodegradation and metabolism, lipid metabolism, and metabolism of terpenoids and polyketides. Figure S6 in Supplementary Material contains LEfSe plots of differentially abundant predicted functions at level 3 of KEGG Orthology for skin site.

## Discussion

Our results suggest that the main force driving the variability in microbiota composition in dogs is the individual, rather than the breed—hair coat—or the skin site. This is true both considering the community structure (weighted UniFrac), but mainly when looking at the less abundant species (unweighted UniFrac). Several human studies have reported that interindividual variation is high and defines a “personal microbiome” (9, 19, 36). These low abundant bacterial signatures have been even used to identify individuals (37).

Meason et al. found recently this same pattern for canine skin mycobiome (fungal community) (17). Moreover, Rodrigues-Hoffmann et al. observed great differences on individuals, although they focused on detecting skin site differences and not on assessing the effect of the individual directly (6). On the other hand, human skin has three main microhabitats or ecological niches, depending on the physiological properties: sebaceous, dry, and moist areas and different microbiota is associated with each microhabitat (5). Conversely, dogs present almost all their skin sites covered by hair that creating a more uniform habitat.

Previous research had detected Proteobacteria (6, 7) or Firmicutes (38) as the main phyla inhabiting dog skin microbiota. Our results suggest that either Proteobacteria or Firmicutes or a combination of both can be the main phyla, depending on the individual. We also found Fusobacteria as one of the most abundant phyla for three of nine dogs, and when it was present, it spread over all the skin sites. Rodrigues-Hoffmann et al. detected Fusobacteria as one phylum specific to perianal regions (6); other studies also found them in groins (8) and paws and forehead (7), but with lower abundances than those seen here.

At the family level, taxa found in our cohort resemble those found in other canine skin microbiota studies (6–8, 18). Rodrigues-Hoffmann et al. found that *Oxalobacteraceae*, specifically *Ralstonia* spp., was the most abundant and extended taxa on dog skin (6), specially on healthy dogs; however, none of our *Oxalobacteraceae* sequences were from *Ralstonia* spp. Pierezan et al. have suggested that this could be due to the use of different supplies for the collection of samples, modifications in sample storage, extraction methods, and/or changes in the high-throughput sequencing platform used (8). *Ralstonia spp*. had been also detected in “blank” controls in microbiota studies and could be contaminants from the laboratory or the kits and reagents used (39).

Among all the individuals included, WH3 was very different with its skin mostly inhabited by *Streptococcaceae* that suggesting a colonization event. The representative sequence of the most abundant *Streptococcaceae* OTU in WH3 corresponds to *Streptococcus canis*, which are considered opportunistic pathogens inhabiting healthy dog skin. Their overgrowth has been associated to dermatitis (40) and even necrotizing fasciitis (41). Moreover, WH3 was the less diverse individual. Low alpha diversity values were characterizing skin microbiota in dogs affected by atopic dermatitis (6, 18), and in humans, they had been linked to elderly people (42). Therefore, we have two hypotheses for WH3: although considered healthy by the clinicians, the dog was beginning to develop some skin affliction; or the effect could be due to its advanced age. Further studies would be needed to assess the effect of age on healthy dog skin microbiota. Reanalyzing results excluding this sample have shown similar results for both ANOSIM and adonis tests (data not shown), confirming that the individual is the main force driving microbiota structure and composition and that the inclusion of this dog does not interfere with the results obtained.

Despite the major force driving microbiota composition and variability was the individual, skin site also plays a role explaining the variability observed. The variability of the skin microbiota regarding the site could be due to the influences of other body site microbiota, such as the perianal region with the gastrointestinal microbiota or the chin with the oral microbiota, or due to the specific physiological properties of each skin site, such as the back with higher sebum production. Perianal region presented the most different composition profile: Bacteroidetes followed by Firmicutes and Fusobacteria were the main phyla, whereas Proteobacteria presented lower abundances. Moreover, Erysipelotrichi and Clostridia classes were overrepresented. This phyla pattern and taxa are more similar to that seen on canine gastrointestinal microbiota than that from the skin (20, 43). At the functional level profiling, some metabolic pathways were significantly overrepresented in the perianal region. Swanson et al. detected carbohydrate metabolism as one of the main pathways in intestinal microbiota of dogs, with values similar to those detected here (20) that are differentially higher than the other skin sites included. In chin, the most abundant phyla were Bacteroidetes, followed by Proteobacteria and Firmicutes. Sturgeon et al. have detected those same three phyla as the most abundant ones on canine oral microbiota (44). Moreover GN02 and TM7, two of the lowest abundant phyla, were overrepresented and differentially distributed on that region. These two phyla have been previously detected in canine oral microbiota (45). We also found that *Porphyromonadaceae* and *Fusobacteraceae* are the most abundant families in chin, coinciding with Bradley et al. who detected *Porphyromonas* and *Fusobacterium* (among others) as abundant genera in canine oral microbiome (16). On the other hand, physiological properties of the back skin could be influencing the microbiota function of that region. The dorsal parts of the neck, the trunk, and the tail have larger sebaceous glands than other skin regions (46). Moreover, the dorsal region has the densest hair coat, so a larger number of sebaceous glands associated with the hair follicles (46). Consequently, more sebum is produced than in other skin sites, which is mainly composed of lipid compounds. The higher abundance of this substrate could be explaining the increased lipid metabolism and fatty acid metabolism pathways in microbiota inhabiting back.

Even when our results show that the main force driving skin microbiota structure and composition is the individual, we cannot rule out the influence of the environment and lifestyle. The individual should be understood as the dog, its lifestyle, and its environment. In fact, the chloroplasts sequences that we detected and discarded for the ulterior analysis were not evenly distributed, but more represented in three dogs (Data Sheet S1 in Supplementary Material), suggesting that these individuals had a greater or more recent exposure to outdoor environment and may have more transient bacterial members detected as skin microbiota. On the other hand, despite being the human skin constantly exposed to extrinsic factors, healthy adults have shown to maintain their skin microbial communities over time (36). This last hypothesis should be assessed in dogs, because they are exposed to extrinsic factors, such as environment or human contact. In this study, we cannot distinguish whether this individual factor is solely host specific or it also includes extrinsic properties from the environment.

Some of the differences when comparing our results to previous studies could be due to differences in the methodologies chosen such as the 16S region analyzed or the sequencing platform used. We are amplifying 400 bp of the V1–V2 hypervariable regions that had been suggested to be a better choice for skin microbiota in humans among others (47). Hypervariable regions V1–V3 are the most commonly used on dog skin microbiota studies (6, 18), but only V2 region has also been used (7). Recently, Pierezan et al. used V4 (8). On the other hand, Clooney et al. found that the factor responsible for the greatest variance in microbiota composition was the chosen methodology, when comparing Illumina HiSeq, Illumina MiSeq, and Ion Torrent PGM. This problem was larger in Illumina MiSeq rather than in Ion Torrent PGM when analyzing 16S rRNA V1–V2 region amplicons (48). In another study comparing microbial profiles using V1–V2 regions, the authors concluded that the output generated from PGM Ion Torrent and 454 yielded concurrent results (49). Finally, PICRUSt is a tool that was mainly developed for the human microbiome. However, dogs share skin microbiota with their owners [as seen in Ref. (7)]. So, using PICRUSt for skin in pets is probably a valid approach. Moreover, PICRUSt has already been used in fecal samples of dogs (21).

## Conclusion

The individual seems to be the main force driving skin microbiota composition and diversity in dogs, and dissimilarity is mainly explained by low-abundant species in each dog. The main phyla inhabiting the dog skin in our cohort are Proteobacteria, Firmicutes, Fusobacteria, Actinobacteria, and Bacteroidetes, and their abundance patterns differ among individuals.

The skin site also plays a role: the composition and function of microorganisms inhabiting chin and perianal region could be influenced by other body site microbiota. Moreover, the specific physiological properties of the back, with higher abundance of sebum, could favor the growth of specific microorganisms. We observed distinctive taxa composition profiles for each sample, but relative abundances become more uniform when converting them to predicted functions.

As the diversity among individuals is the highest, a good choice to better assess the dog skin microbiota would probably be comparing affected vs unaffected regions from the same dog rather than comparing different dogs in case–control studies, so each dog is its own control; and an accurate assessment of the environmental factor, controlling variables such as geographical region, season, lifestyle, or cohabitation with other animals.

## Author Contributions

AS, LF, and OF conceived and designed the experiment. AS, LA, LF, and OF supervised the project and gave conceptual advice. AC and OF performed the experiment. AC carried out the bioinformatics analysis. AC drafted the manuscript. AS, LA, LF, and OF edited the manuscript. All authors read and approved the final manuscript.

## Conflict of Interest Statement

The authors declare that the research was conducted in the absence of any commercial or financial relationships that could be construed as a potential conflict of interest.
