# Corrigendum: Individual Signatures Define Canine Skin Microbiota Composition and Variability

**DOI:** 10.3389/fvets.2017.00119

**Published:** 2017-07-28

**Authors:** Anna Cuscó, Armand Sánchez, Laura Altet, Lluís Ferrer, Olga Francino

**Affiliations:** ^1^Molecular Genetics Veterinary Service (SVGM), Veterinary School, Universitat Autònoma de Barcelona, Barcelona, Spain; ^2^Vetgenomics, Ed Eureka, Parc de Recerca UAB, Barcelona, Spain; ^3^Department of Clinical Sciences, Cummings School of Veterinary Medicine, Tufts University, North Grafton, MA, United States

**Keywords:** skin, microbiota, microbiome, dog, canine, coat, skin site, 16S

In the original article, there was a mistake in Figure 3 as published. Although Figure 3 is scientifically meaningful, it does not fit the legend and the associated text on the manuscript. The corrected Figure 3 appears below. The authors apologize for this error and state that this does not change the scientific conclusions of the article in any way.

**Figure 3:**
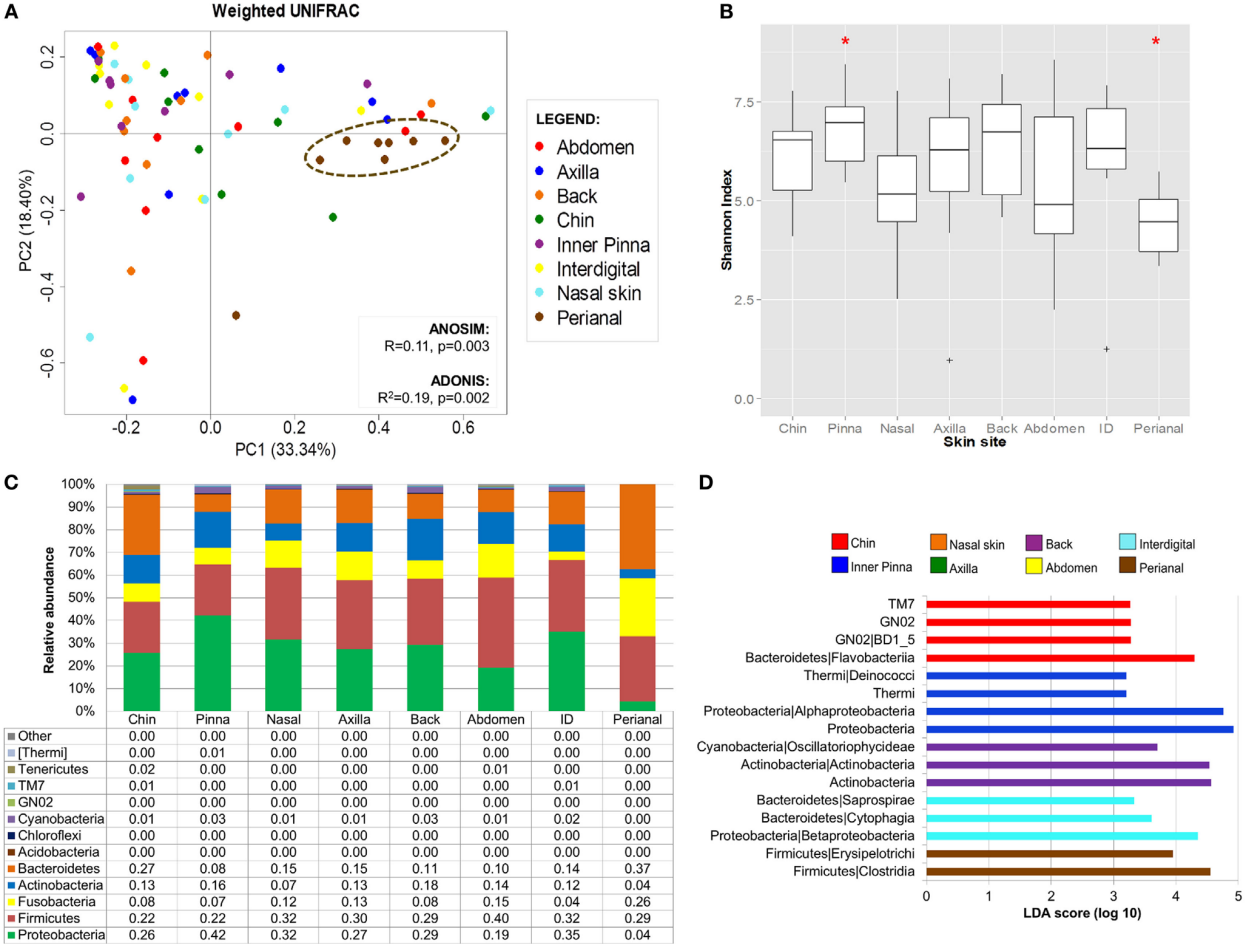
Dog skin microbiota analysis considering site. **(A)** PCoA plot using weighted UniFrac metrics colored by skin site with values of ANOSIM and adonis statistical tests. Perianal region is circled in brown. **(B)** Boxplots of alpha diversity values. Marked with a red asterisk the two comparisons that were statistically different when using Monte Carlo permutation test (*P* < 0.05). **(C)** Bar plot representing skin microbiome composition at phylum level per skin site; each bar represents the mean values of the nine dogs per each skin site. **(D)** Histogram of linear discriminant analysis (LDA) effect size scores for differentially abundance distribution (α = 0.05, LDA score >3) of bacterial phyla and classes among skin sites.

## Conflict of Interest Statement

The authors declare that the research was conducted in the absence of any commercial or financial relationships that could be construed as a potential conflict of interest.

