# Supplementary material for: Individual Signatures Define Canine Skin Microbiota Composition and Variability
Source: Front Vet Sci. 2017 Feb 6;4:6. doi: 10.3389/fvets.2017.00006 (PMC5292769; doi:10.3389/fvets.2017.00006)
Supplement: Supplementary file 1 [file Presentation_1.PDF]

Supplementary Material

Individual Signatures Define Canine Skin Microbiota Composition and Variability

Anna Cuscó*, Armand Sánchez, Laura Altet, Lluís Ferrer, Olga Francino

*** Correspondence:** Anna Cuscó: anna.cusco@vetgenomics.com

1 Supplementary Data

Supplementary Data S1. Number of sequences per sample in both open and closed reference approaches. [a]Initial sequences are basic quality-filtered sequences included at OTU table before extra filtering steps. Extra filtering steps discard first sequences belonging to chloroplasts class[b] and then low-abundant OTUs[c] to obtain the valid sequences[d] used for downstream analyses. (.xlsx 23KB).

Supplementary Data S2. Operational taxonomic units (OTU) table rarefied at 25,000 sequences per sample. Complete OTU table (A): numbers represent absolute read counts per each OTU and sample. OTU table up to family level with all samples (B), samples collapsed by site (C) and samples collapsed by individual (D): relative abundances of each OTU at phylum (bold and capital letters) and family level per sample. (.xlsx 748KB).

Supplementary Data S3. Alpha diversity values at depth of 25,000 sequences per sample. Values of (A) each single sample and (B) average values considering skin site, skin site per breed and breed at 25,000 sequences per sample. (.xlsx 17 KB)

Supplementary Data S4. Skin core microbiome (at family level) for every individual and skin site. Taxa present in 61 out of 71 skin samples (85% of the samples). (.xlsx 17 KB)

Supplementary Data S5. Mean relative abundances of predicted functions at depth of 10,000 sequences per sample. Abundances of (A) all samples and (B-C) skin sites (average values). Predicted functions were performed at the second and third level of KEGG Orthology. (.xlsx 289 KB)

2 Supplementary Figures and Tables

2.1 Supplementary Figures

Supplementary Figure S1. Alpha diversity rarefaction curves. Alpha diversity rarefaction curves per individual (a, b) and skin site (c, d) using Observed species and Shannon Index.

a.

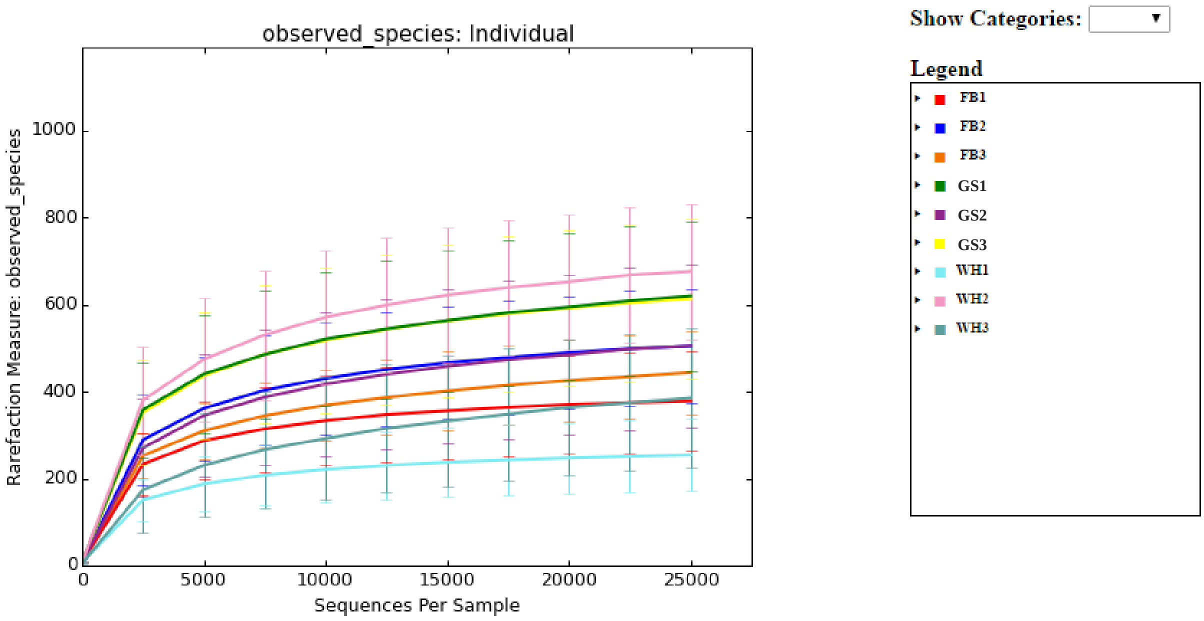

b.

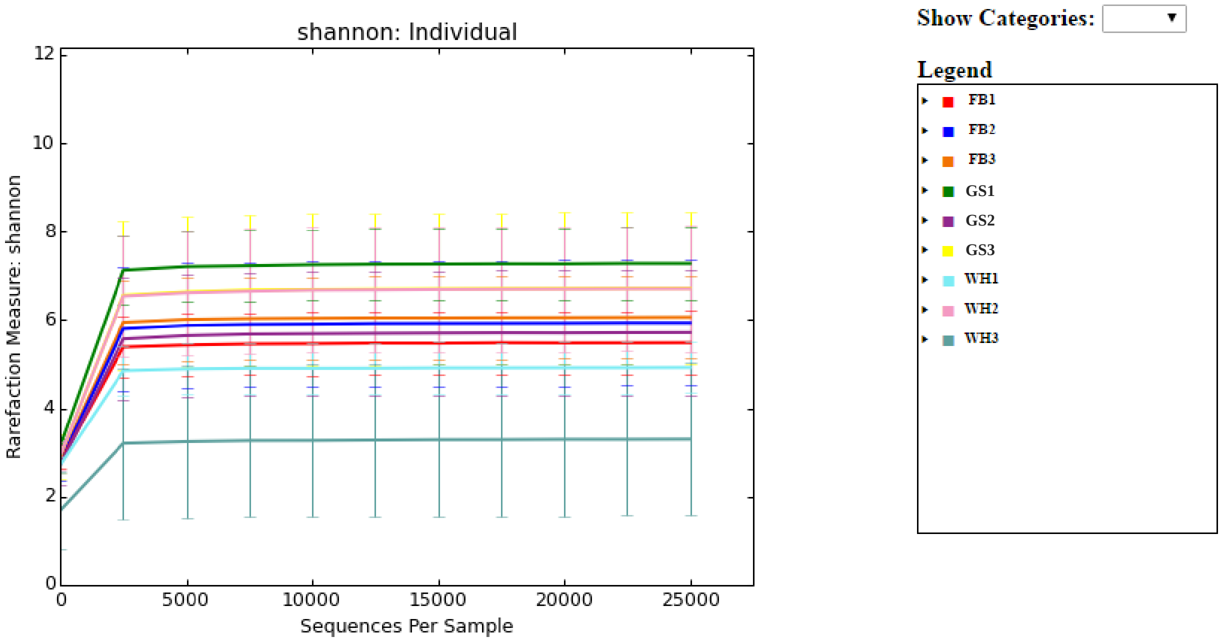

c.

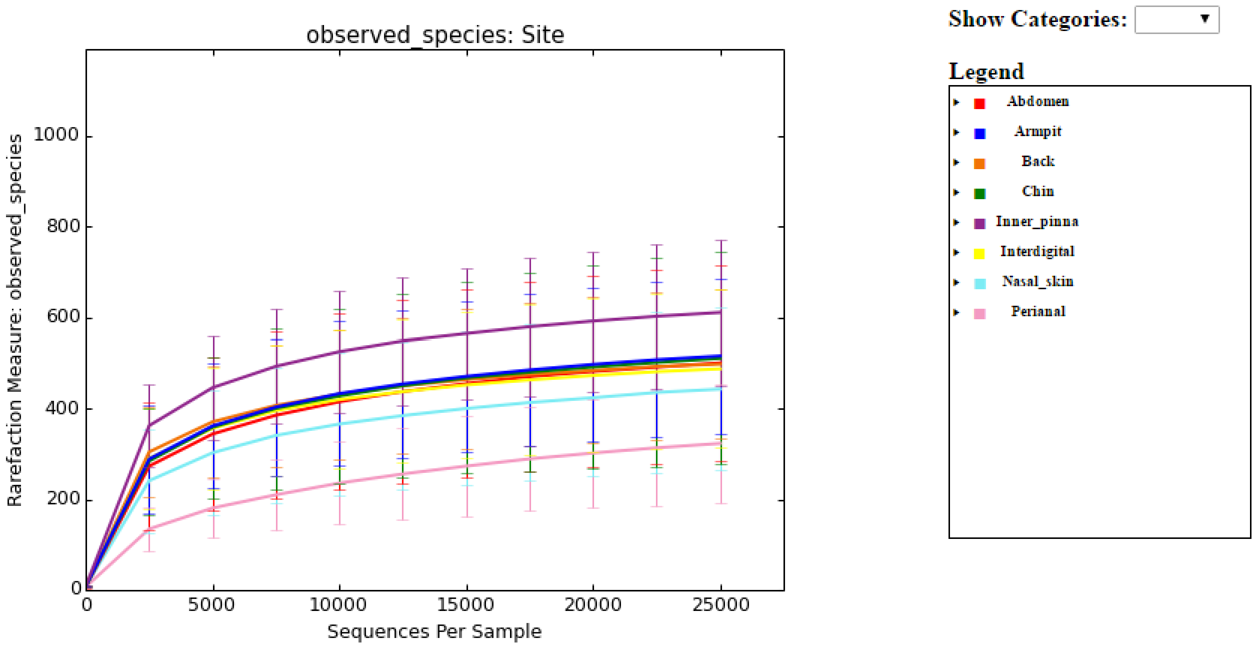
observed_species: Site
Rarefaction Measure: observed_species
Sequences Per Sample
0
200
400
600
800
1000
0
5000
10000
15000
20000
25000
Show Categories:
Legend
Abdomen
Armpit
Back
Chin
Inner_pinna
Interdigital
Nasal_skin
Perianal

d.

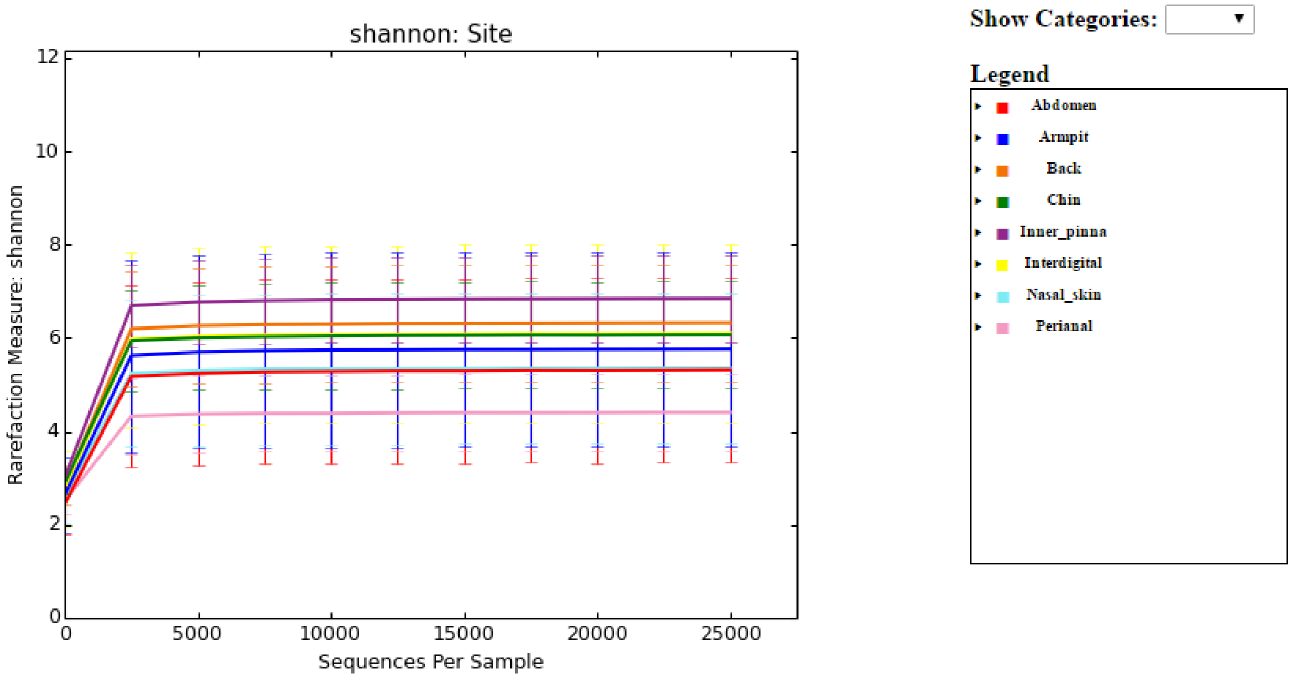
shannon: Site
Rarefaction Measure: shannon
Sequences Per Sample
0
2
4
6
8
10
12
0
5000
10000
15000
20000
25000
Show Categories:
Legend
Abdomen
Armpit
Back
Chin
Inner_pinna
Interdigital
Nasal_skin
Perianal

Supplementary Figure S2. Dog skin microbiota analysis considering skin site. (A) PCoA plot using weighted UNIFRAC metrics colored by skin site with values of ANOSIM and adonis statistical tests. (B) Histogram of Linear Discriminant Analysis (LDA) Effect Size (LEfSe) scores for differentially abundance distribution (α=0.05, LDA score>3) of bacterial phyla among individuals.

a.

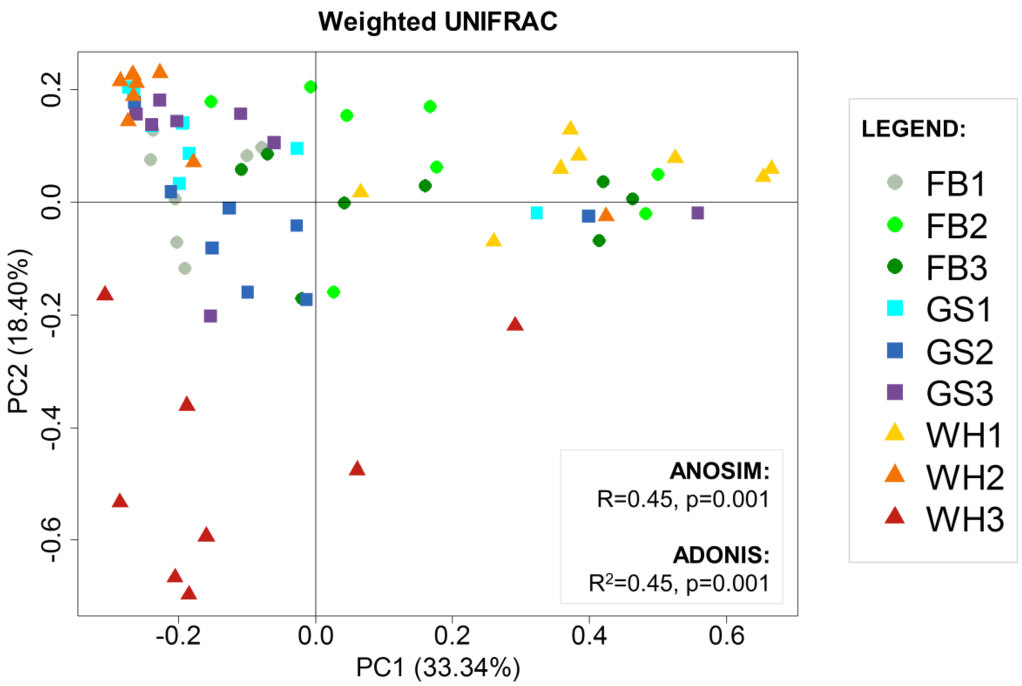

b.

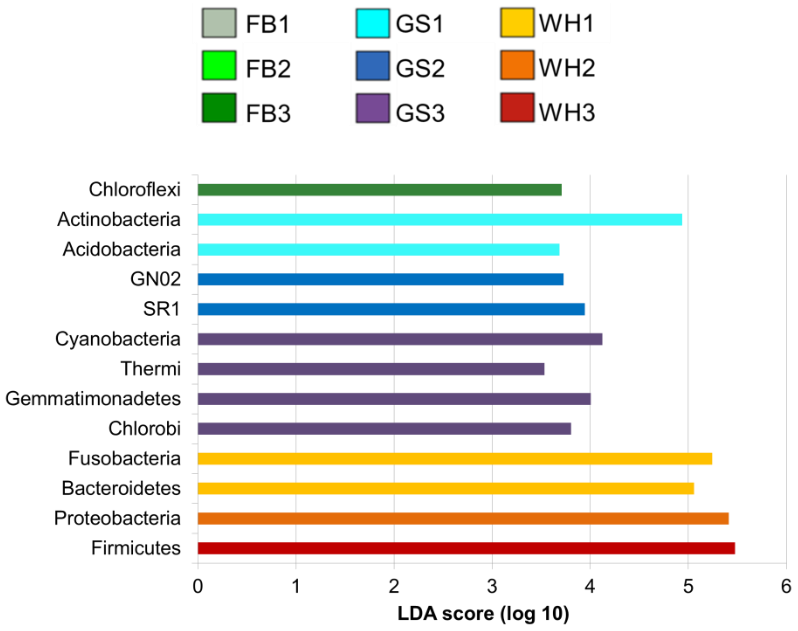

Supplementary Figure S3. Histogram of LDA scores for differentially abundant (α=0.05, LDA score > 3.0) taxa (up to family level) among the different skin sites.

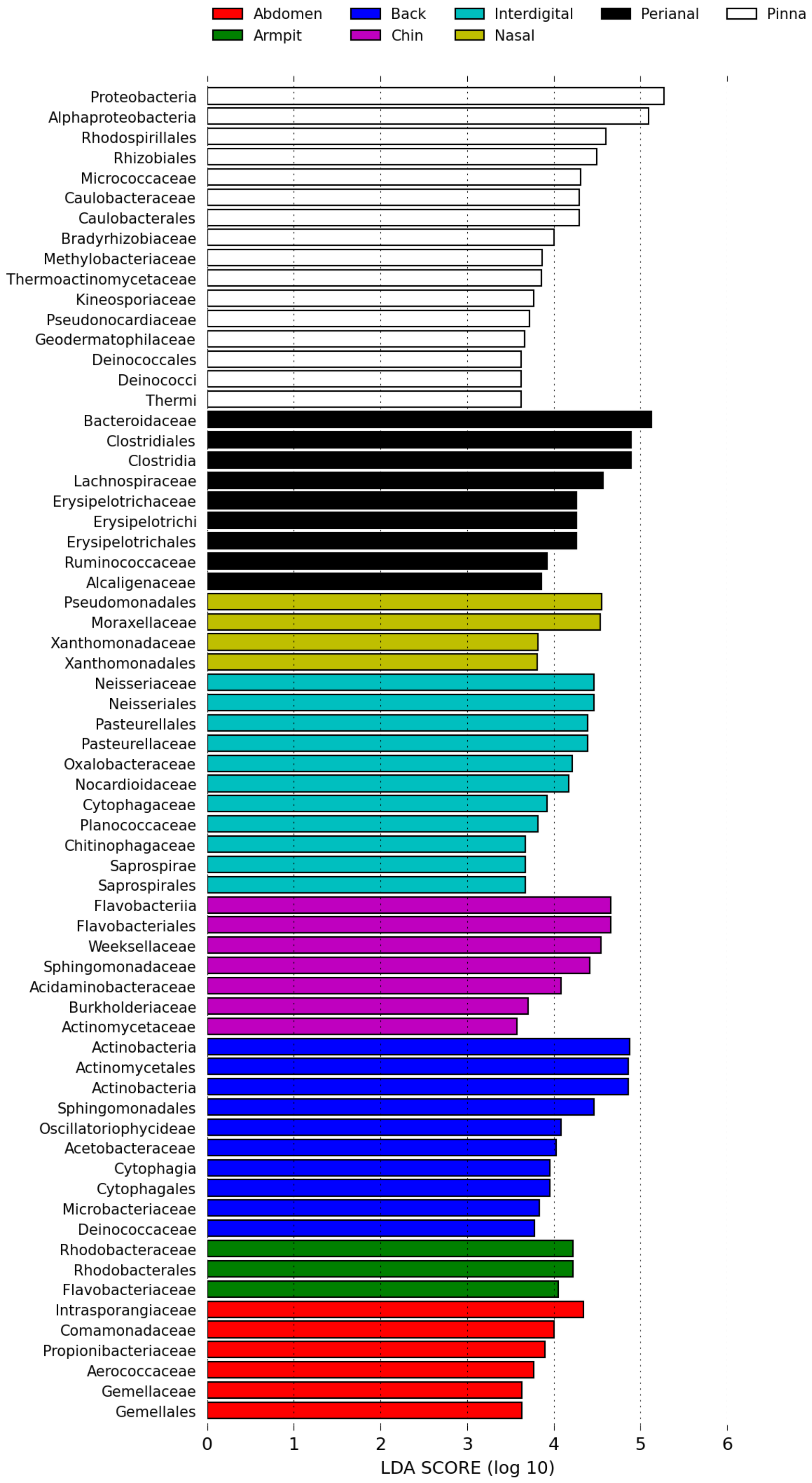

Supplementary Figure S4. Dog skin microbiome analysis considering breed. PCoA plots using unweighted (A) and weighted (B) UNIFRAC metrics colored by breed with values of ANOSIM and adonis statistical tests. (C and D) Histogram of Linear Discriminant Analysis (LDA) Effect Size (LEfSe) scores for differentially abundance distribution (α=0.05, LDA score>3) of bacterial taxa among breeds when considering each skin site independently (C) or when collapsing samples to form a global sample (D) (see materials and methods for more information).

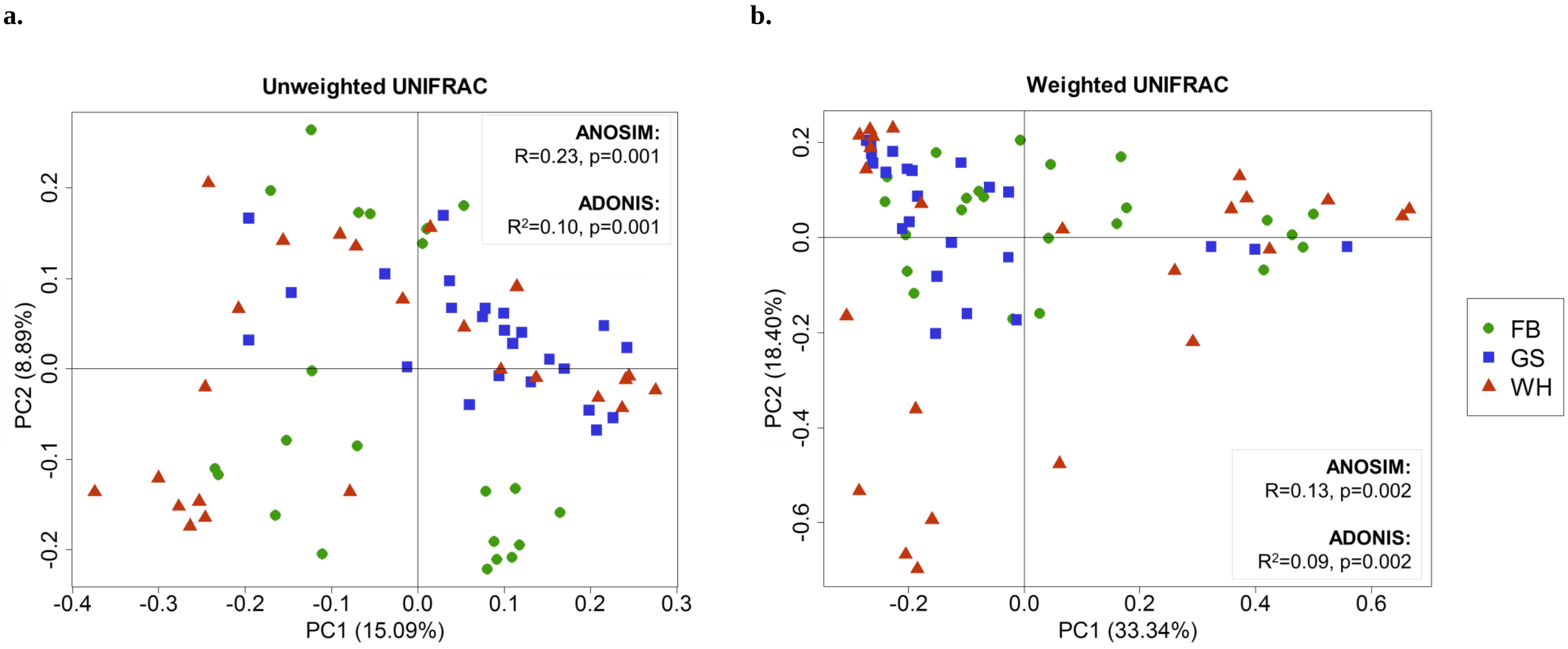

c.

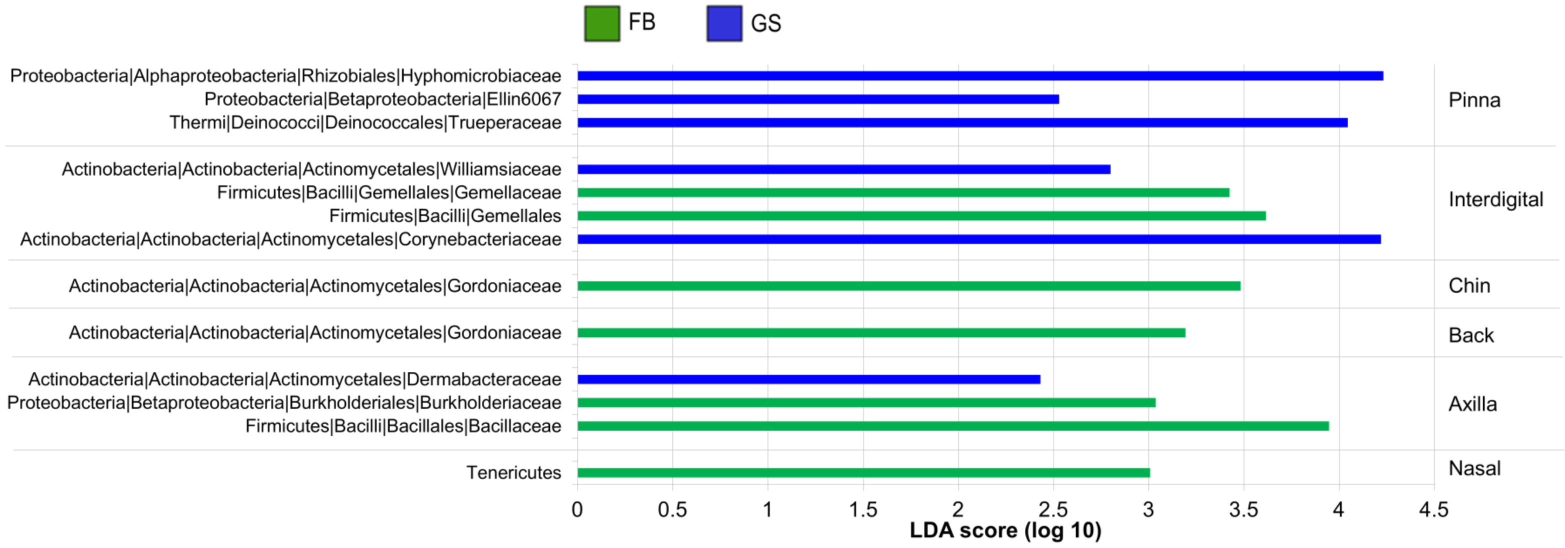
FB
GS
Proteobacteria|Alphaproteobacteria|Rhizobiales|Hyphomicrobiaceae
Proteobacteria|Betaproteobacteria|Ellin6067
Thermi|Deinococci|Deinococcales|Trueperaceae
Pinna
Actinobacteria|Actinobacteria|Actinomycetales|Williamsiaceae
Firmicutes|Bacilli|Gemellales|Gemellaceae
Firmicutes|Bacilli|Gemellales
Actinobacteria|Actinobacteria|Actinomycetales|Corynebacteriaceae
Interdigital
Actinobacteria|Actinobacteria|Actinomycetales|Gordoniaceae
Chin
Actinobacteria|Actinobacteria|Actinomycetales|Gordoniaceae
Back
Actinobacteria|Actinobacteria|Actinomycetales|Dermabacteraceae
Proteobacteria|Betaproteobacteria|Burkholderiales|Burkholderiaceae
Firmicutes|Bacilli|Bacillales|Bacillaceae
Axilla
Tenericutes
Nasal
0 0.5 1 1.5 2 2.5 3 3.5 4 4.5
LDA score (log 10)

d.

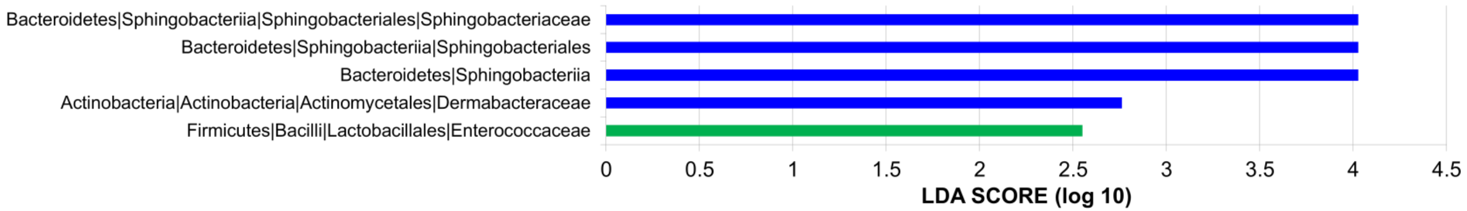
Bacteroidetes|Sphingobacteriia|Sphingobacteriales|Sphingobacteriaceae
Bacteroidetes|Sphingobacteriia|Sphingobacteriales
Bacteroidetes|Sphingobacteriia
Actinobacteria|Actinobacteria|Actinomycetales|Dermabacteraceae
Firmicutes|Bacilli|Lactobacillales|Enterococcaceae
0 0.5 1 1.5 2 2.5 3 3.5 4 4.5
LDA SCORE (log 10)

Supplementary Figure S5. Boxplots representing alpha diversity values when assessing the variable breed. We considered two approaches: (a) analyzing each skin site independently and (b) collapsing eight skin samples to form an individual sample No statistically significant differences were detected. Each boxplot is representing a sample size of n=3 (see material and methods for details).

a.

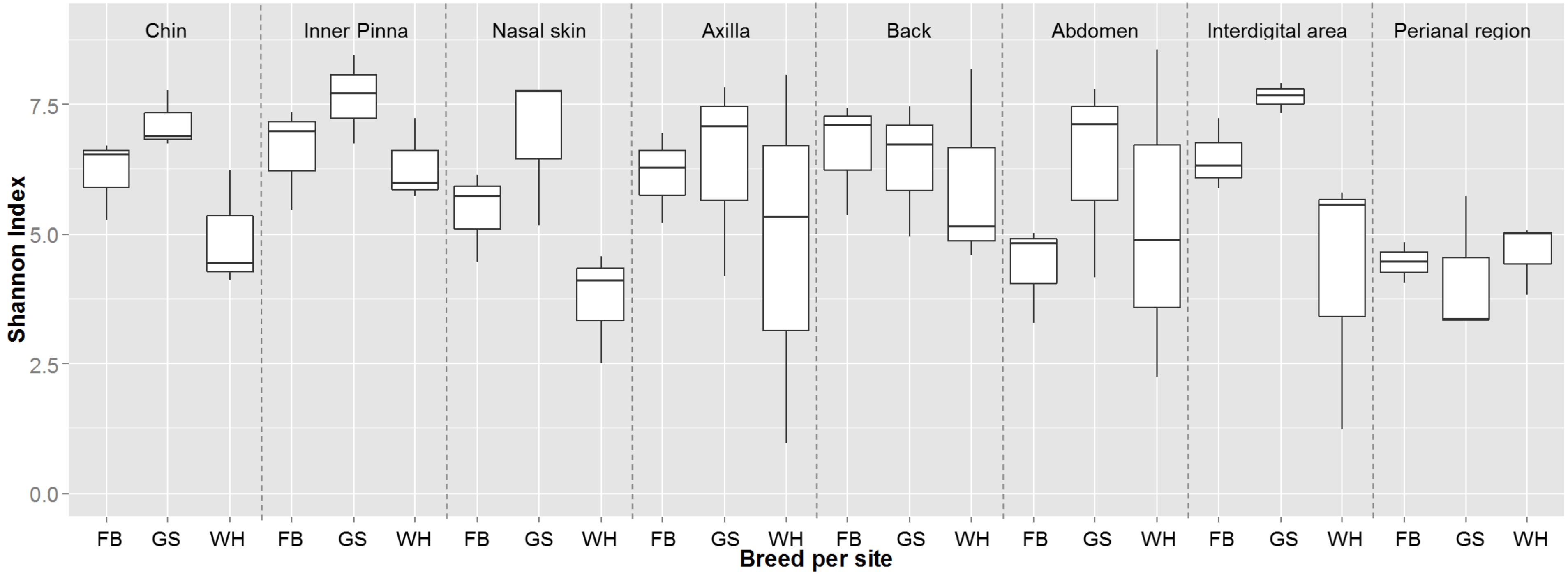

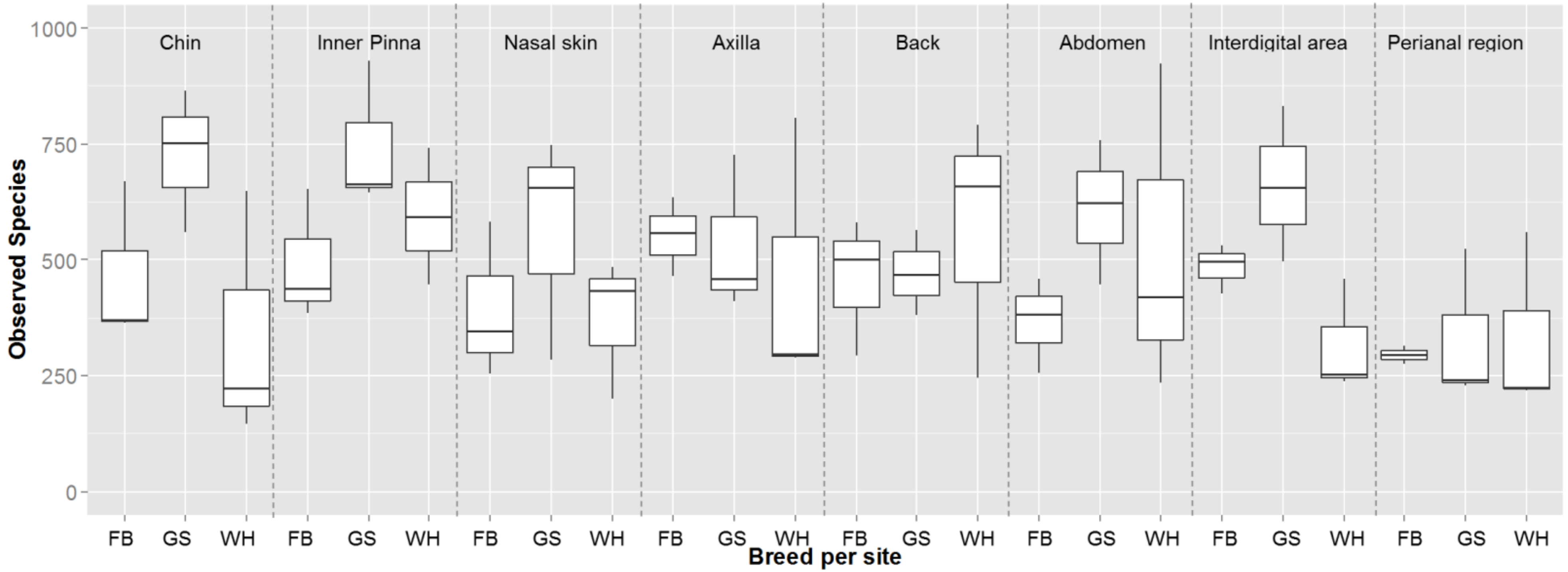

Chin
Inner Pinna
Nasal skin
Axilla
Back
Abdomen
Interdigital area
Perianal region
1000
750
500
250
0
Observed Species
FB GS WH FB GS WH FB GS WH FB GS WH FB GS WH FB GS WH FB GS WH FB GS WH
Breed per site

b.

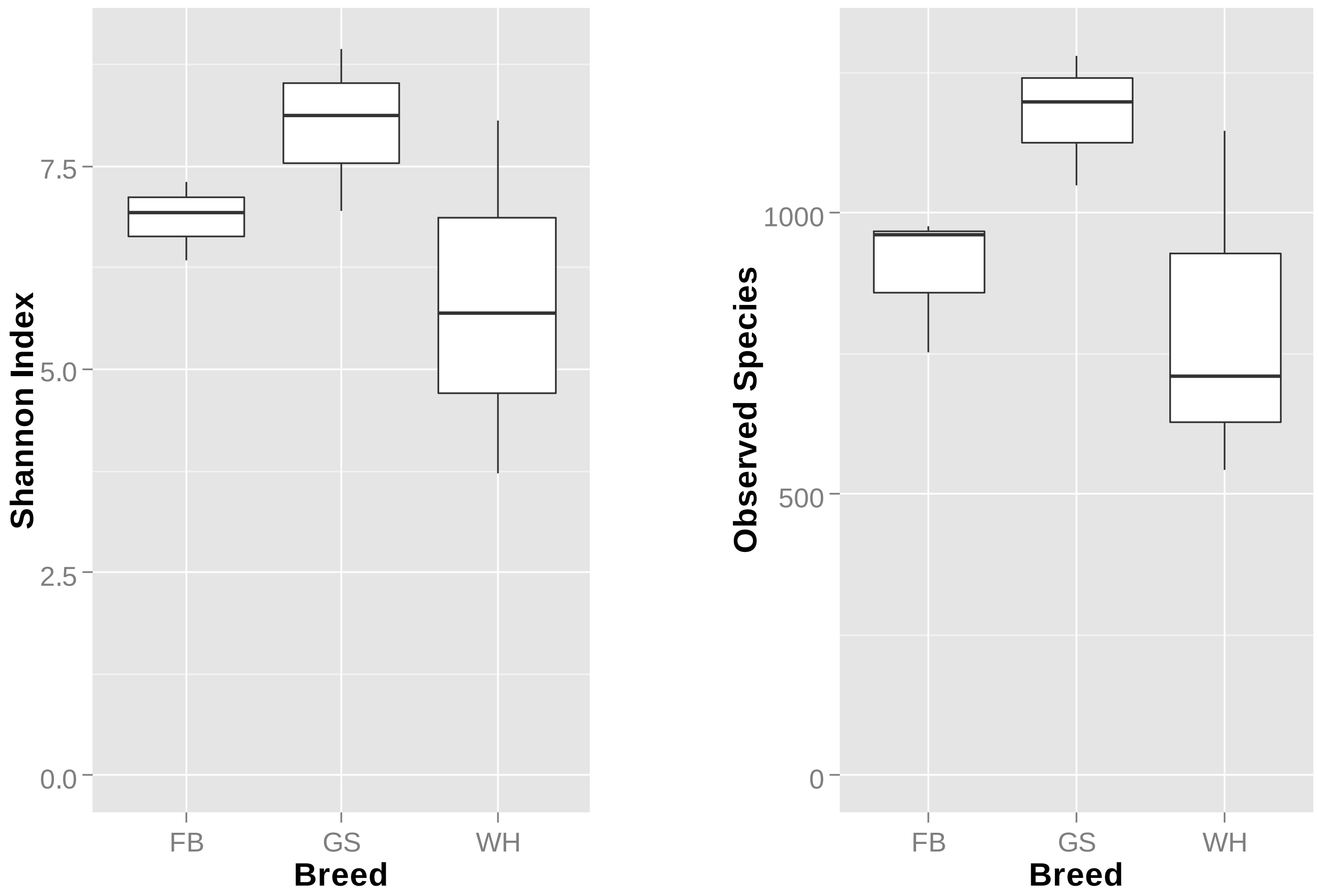

Shannon Index
7.5
5.0
2.5
0.0
FB
GS
WH
Breed
Observed Species
1000
500
0
FB
GS
WH
Breed

Supplementary Figure S6. Histogram of LDA scores for differentially abundant (α=0.05, LDA score > 3.0) functions among the different skin sites.

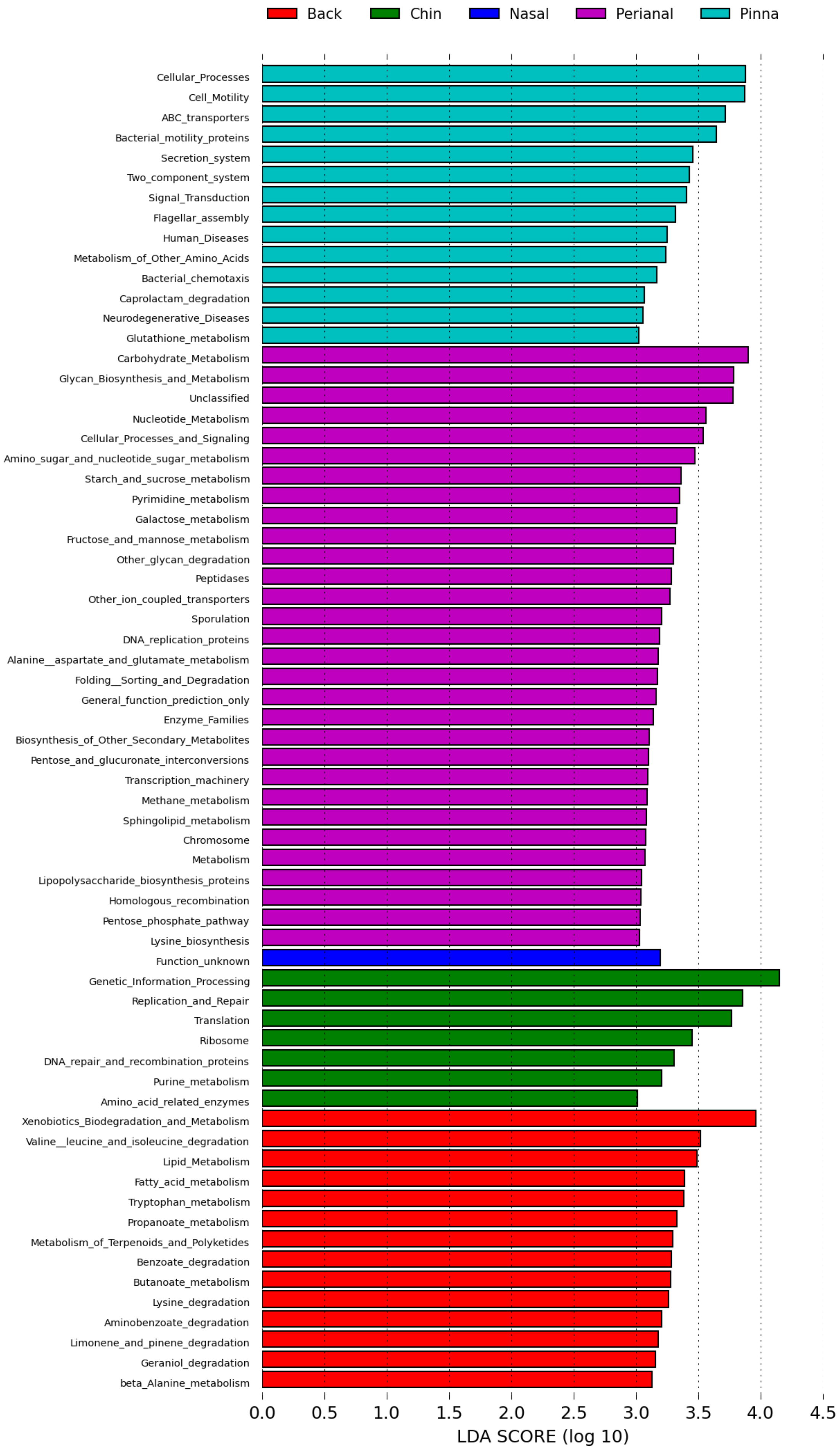

2.2. Supplementary Tables

Supplementary Table S1. Information of the dogs included on the study.

Dog ID	Breed	Sex	Age
FB1	French bulldog	Female	2 years old and 5 months
FB2	French bulldog	Male	9 months
FB3	French bulldog	Male	7 years old
WH1	West Highland white terrier	Male	6 years old and 5 months
WH2	West Highland white terrier	Male	3 years old and 3 months
WH3	West Highland white terrier	Female	12 years old
GS1	German shepherd	Male	3 months
GS2	German shepherd	Male	10 months
GS3	German shepherd	Female	9 years old
